# Engaging in moral learning: veterans’ perspectives on how the moral dimensions of moral injury are addressed in one-on-one meetings with Dutch military chaplains

**DOI:** 10.3389/fsoc.2025.1488372

**Published:** 2025-02-21

**Authors:** Laura Mudde, Carmen Schuhmann, Gaby Jacobs

**Affiliations:** University of Humanistic Studies, Research Group Humanist Chaplaincy Studies for a Plural Society, Utrecht, Netherlands

**Keywords:** moral injury, veterans, military chaplaincy, moral learning, longitudinal research

## Abstract

**Introduction:**

There is an increasing attention for the role of military chaplains (MCs) in supporting veterans with moral injury. However, research into how veterans experience the support of MCs remains scarce. Moreover, no studies to date have explored this question in a Dutch contex, while this is relevant as it can offer insight into what forms of care are helpful in predominantly secular societies.

**Methods:**

This article presents a study from the Netherlands, involving 12 veterans. Using a longitudinal qualitative approach, we explored how the one-on-one conversations with MCs unfold over time.

**Results:**

Our study shows that three types of moral questions underly experiences of moral injury. Veterans see the conversations with MCs as an opportunity to exchange thoughts and perspectives concerning these ongoing moral struggles, a process that we conceptualize as ‘moral learning’. Over time, we found 5 types of change in veterans’ experience of moral injury. The conversations with MCs helped veterans to: share their stories, thoughts and worries; grow personally; better understand and accept certain events; feel a stronger connection with others; critically engage with the Dutch Ministry of Defence.

**Discussion:**

This study raises questions about the centrality of the morally injurious events in chaplaincy interventions that are described in the literature. It suggests that supporting veterans in dealing with questions about the good life and about the conduct of the military may be just as or even more important as reflecting on morally injurious events. Moreover, the study highlights the importance of engaging with seemingly mundane, everyday issues when addressing the moral dimensions of veterans’ struggles. This counters the focus on grand concepts like “forgiveness,” “acceptance,” “reconciliation,” “restitution” and “vindication” which are usually emphasized in the literature about chaplaincy in the context of moral injury. The study shows that it is through reflection on the everyday that these larger concepts gain relevance and meaning within veterans’ lives.

## Introduction

Since Shay (1994) introduced the concept of moral injury, and Litz et al. (2009) brought it to broader interdisciplinary attention, research into the moral injury of veterans has expanded rapidly. While the debate continues over its precise definition and the theoretical models that explain its occurrence (or absence) (Griffin et al., 2019; Ter Heide, 2020; Koenig and Al Zaben, 2021), there is general agreement that moral injury is a complex phenomenon encompassing psychological, social, and existential (spiritual and moral) dimensions (Jinkerson, 2016; Litz and Kerig, 2019; Litz et al., 2009). Moral injury is commonly defined as the lasting and profound struggles experienced by individuals following situations where they perpetrate, fail to prevent, witness, or learn about actions that violate deeply held moral beliefs and expectations (Litz et al., 2009). The literature on moral injury reflects two perspectives, which do not exclude each other but point to a difference in focus. On the one hand, there are the accounts that focus on the internal, individual experiences of moral injury as a deeply personal struggle, building on the conceptualization that was brought forward by Litz et al. (2009).

On the other hand, some authors emphasize the sociopolitical dimensions of moral injury, framing individual struggles as inherently tied to broader societal and institutional contexts (Molendijk, 2018, 2022) and underline that these moral struggles can offer valuable insights into societal norms and conducts (Kinghorn, 2012; Antal, 2017; Wiinikka-Lydon, 2018, 2022). Our study aligns with this second perspective. While moral injury may co-exist with Post-traumatic stress disorder (PTSD), depression, anxiety, and suicidal thoughts (Barnes et al., 2019; Koenig and Al Zaben, 2021), we emphasize that experiencing moral injury is not inherently pathological.

We approach moral injury as a reasonable and justified moral response to transgressions of deeply held moral beliefs and identities that constitute a person. The result of such events is unwanted dissolutions of these moral beliefs and identities, which leads to significant psychological, social, and existential-spiritual suffering. As moral injury arises from transgressions of what is deemed right, it is crucial to address the specific moral dimensions of these experiences when supporting veterans with moral injury (Jones et al., 2022; Kopacz et al., 2016; Koenig and Al Zaben, 2021; Shay, 1994; Williamson et al., 2021).

The role of military chaplaincy (MC) has been highlighted in this regard (Carey et al., 2016; Carey and Hodgson, 2018; Doehring, 2019; Kopacz et al., 2019; Meador and Nieuwsma, 2018). However, research into how veterans experience MC support remains scarce (Harris et al., 2018; Jones et al., 2022). Existing empirical studies on how MCs address moral injury either examine the issue from the perspective of chaplains (Carey et al., 2023; Chang et al., 2015; Drescher et al., 2018; Grimell, 2023a; Pleizier and Schuhmann, 2022) or focus on specific chaplaincy interventions that are designed for aiding veterans who experience moral injury. These are, for example, the *Warrior’s Journey intervention* (Fleming, 2020), *Pastoral Narrative Disclosure* (Carey and Hodgson, 2018), *Building Spiritual Strength* (Usset et al., 2021), and *Acceptance and Forgiveness Therapy* (Pernicano et al., 2022). Empirical studies about these interventions generally aim to measure the effectiveness of interventions in alleviating symptoms of moral injury, mostly by employing quantitative methods (Jones et al., 2022). There is still a need for rich, qualitative insights into veterans’ experiences, particularly through longitudinal research (Koenig and Al Zaben, 2021).

Therefore, this study aims to shed light on how one-on-one conversations with MCs support veterans in dealing with their moral struggles. Moreover, no studies to date have explored the experiences of Dutch veterans who engage in one-on-one meetings with MCs (Jones et al., 2022). We believe that researching the experience of Dutch veterans with regard to the moral dimension of their struggles is relevant, as it can offer insight into what forms of care are helpful in predominantly secular societies. Therefore, the central question that we address in this article is: How do Dutch post-active veterans with moral injury perceive the value of one-on-one conversations with MCs regarding their moral struggles?

### Dutch veteran population

According to the latest Veteran Report from the Dutch Ministry of Defence (2023),^1^ there are currently 100,800 Dutch veterans, of whom 76,000 are no longer in active service. Among these post-active veterans, 93.3% are men, and 6.7% are women. Their average age is 53.2 years. The Dutch Ministry of Defence does not collect data on its personnel’s ethnicity or religious backgrounds, so no detailed information is available in these areas. To still give some idea about the make-up of the general Dutch population in this regard: in recent decades, religious involvement in the Netherlands has steadily declined, and the country is now considered predominantly secular (Huijnk and Van Houwelingen, 2022). While 43% of the Dutch population identifies as belonging to a religious or spiritual community, most of these people do not actively practice their religion, as only 12% regularly attend religious services.^2^

### General well-being of Dutch veterans

A large study conducted by the Netherlands Veteran Institute in 2021 involving 3,142 post-active veterans sheds light on their well-being. The study found that 15% of Dutch post-active veterans experience personal problems that are, at least in part, related to their participation in military missions. These problems are primarily psychological (71%) and relational (41%). Among this group, 40% do not seek professional help. These veterans express a desire to resolve their issues independently, lack motivation, or have negative past experiences with professional care. Approximately half of the post-active veterans seeking help receive it through the National Healthcare System for Veterans [Landelijk Zorgsysteem voor Veteranen (LZV)] (Duel et al., 2022).

Research into the care provided by the LZV highlights the complexity and enduring challenges faced by veterans. While veterans generally report positive experiences with the care they receive, their evaluations of their overall well-being and recovery progress are predominantly negative (Duel and Vlaar, 2024, p. 19–20). More than half of the veterans who receive professional support have been in treatment for over a year. Nevertheless, slightly more than half of this group rate their well-being as poor or insufficient. Furthermore, 48% have reported no improvement in their well-being over the last 3 months of treatment, and 25% have experienced a worsening of their condition during this period (Duel and Vlaar, 2024, p. 8). A fact sheet^3^ from the Netherlands Veteran Institute, which compiles data from various studies, indicates that the incidence of PTSD among Dutch veterans varies by mission, ranging from 2 to 9% (see also Bramsen et al., 2000). Currently, no statistics are available on the prevalence of moral injury within the Dutch veteran population.

In 2022, the Professional Guideline on Moral Injury was developed by a multidisciplinary working committee comprising a clinical psychologist, specialist social worker, health psychologist, humanist chaplain, and an expert by experience (Ter Heide et al., 2022). This guideline is now employed within the LZV to facilitate a more comprehensive understanding of the disparate approaches to moral injury among the various professional fields of social work, chaplaincy, and trauma therapy and to delineate the roles of support for veterans who experience moral injury. As moral injury is not a clinical diagnosis in the Netherlands, it does not qualify veterans for financial compensation, in contrast to the diagnosis of PTSD. However, the development of this professional guideline demonstrates an increasing recognition of moral injury as a distinct and pressing concern among Dutch veterans.

### Conceptualization of moral learning: a Deweyian perspective

In this study, we adopt the viewpoint that moral struggles are inherent to life and propose that dealing with moral injury can be conceptualized as ‘moral learning.’ We reached this conceptualization through a pragmatist lens, particularly drawing on John Dewey’s works: *Human Nature and Conduct* (1922), *Theory of the Moral Life* (1932), *Experience and Education* (1938a), and *Logic, the Theory of Inquiry* (1938b). While Dewey’s writings have not previously been associated with moral injury, we find that his reflections on into morality and learning provide a deeper understanding of the patterns of change observed in the lives of veterans.

### A social and situated outlook on morality

Pragmatist epistemology is predicated on the notion that there is no objective reality external to our perception. Instead, our interactions with the social and physical world shape our understanding of reality. In light of this, a pragmatist perspective on morality is predicated on the notion that there is no such thing as an absolute moral good or a fixed set of moral rules to be adhered to (Heney, 2022). Furthermore, it is a descriptive approach that begins with an examination of the lived experiences of actual individuals (Addams, 2007). Morality is seen as inherently socially constructed (Dewey, 1922, 1932; Mead, 1923). It *emerges* from embodied and socially embedded activities as individuals engage with their environment (Dewey, 1922, 1932). Our sense of morality is thus shaped by our interaction with the social and physical world, and values are, therefore, necessarily time- and context-dependent. We determine what is good through a relational and imaginative exploration of how to harmonize competing goals and enrich meaning in problematic situations (Johnson, 2019, p. 177). This approach to morality allows us to interpret (everyday) events, relations, and interactions as moral learning processes.

### Experiential and transformative moral learning

Learning then, is conceptualized by Dewey as an experiential process in which people interact with their environment, take in new experiences, respond to them, and assign meaning to them (English and Doddington, 2019). Moreover, these learning processes are always understood as transformative processes. According to Dewey, our sense of self is shaped by our engagement with the social and physical environment. There is no sense of self without action in the world (Dewey, 1932, pp. 151–151). Therefore, when we learn new ways of interacting with or seeing the world, we not only acquire new meaning about the world but also transform ourselves (Dewey, 1938a). Here, Dewey has a different stance than contemporary writers about transformative learning, such as Mezirow (1978, 2008), who conceptualizes transformative learning as a specific type of learning, demarcated from other forms of learning (Dirkx, 1998).

The concept of habits – ‘*the art of doing things’* (Dewey, 1922, p. 15) - plays a central role in Dewey’s understanding of learning as transformative processes. Habits are the ‘*medium’* through which every experience of the world gets ‘*filtered*’ (*Ibid*, p. 25). By shaping our experiences, our habits constitute our selfhood (Dewey, 1922, p. 58–75). The concept of habits, therefore, allows us to discern when learning occurred: a change of habits signifies a transformation of someone’s selfhood and, thus, learning. So, to capture processes of moral learning we pay attention to both veterans’ sayings and doings’.

To better understand the nature of learning processes, it is useful to shed light on the role of conflict in Dewey’s writings on both learning and morality (Johnson, 2019; Sund and Öhman, 2023). Dewey writes that a change of morals occurs only through some condition of affairs that has not yet been experienced (Dewey, 1922, p. 56). Such a shift is prompted by a conflict, a situation in which the habits are no longer applicable (Dewey, 1922, 1938b; Miettinen, 2000). This prompts a period of reflection and the initiation of what Dewey terms ‘*impulsive activities’* (Dewey, 1922, p. 87): a process of exploring alternative modes of interaction, perception, and valuation in relation to the situation at hand. However, the potential for impulsive activities to be transformed into new habits is contingent upon the environment. For impulsive activities to gain meaning, they must be met with responsiveness from others. In other words, impulses are merely the initial catalyst for change; they must be integrated into the social context of an individual to gain meaning and be developed into new habits that ultimately alter someone’s selfhood.

In sum, moral learning can be understood as an experiential, relational form of learning within the moral domain. Such learning is prompted by conflict and, with time and responsive reactions of others, changes a person’s moral view of themselves, others, and the world. In the words of Dewey himself, learning about the good is: *“…a learning that takes us back not into an isolated self but into the open-air world of object and social ties, accumulating in an increment of present signification.”* (*Ibid.*, p. 291).

## Method

We employed a longitudinal qualitative research approach to come to a rich, descriptive study of how Dutch post-active veterans with moral injury perceive the value of one-on-one conversations with MCs regarding their moral struggles. The longitudinal design allowed us to track the same veterans over an extended period of time, capturing how their experiences evolved (Thomson and Holland, 2003). We aimed to gain insight into the internal logic of development or change according to the veterans (Calman et al., 2013; McLeod and Thomson, 2009). The research was conducted between May 2022 and July 2024, and data collection spanned from October 2022 until April 2024.

### Participants and selection

The study involved a cohort of 12 post-active veterans.^4^ Veterans were approached through the participating MCs using a snowball approach (Patton, 2014). The first selection criterion was that veterans had indeed experienced moral injury.

Veterans completed the Moral Injury Event Scale (Nash et al., 2013; De Goede et al., 2022) to ascertain the presence of moral injury. Second, we anticipated that not all veterans are willing or in a condition to elaborate on their experiences; we, therefore, asked the participating MCs to bring us in contact with veterans who could provide detailed descriptions of their experiences. Last, since the contact between veterans and MCs often extended beyond the 1.5-year scope of our data collection period, we included both veterans in initial and ongoing contact with MCs to gain a comprehensive view of the one-on-one guidance over different time periods (Table 1).

**Table 1:** Sample of veterans.

Gender	Age	Religious/spiritual outlook	Missions	MIES	Received care
*M* = 12	41–63 years	None = 7 Catholic = 4 Pagan = 1	UNIFIL = 1 UNTAC = 1 UNPROFOR = 6 ECMM = 1 KFOR = 1 ISAF = 5	Moderate = 4 High = 3 Severe = 4 Two veterans had difficulty completing the MIES since the questions were very triggering. The first author gave them the option to not complete the scale.	All participating veterans had received or were receiving therapy for PTSD-related symptoms. Years of receiving care from civil or military care services: 1–8

UNIFIL is the United Nations Interim Force in Lebanon (1978 – present). UNTAC, United Nations Transitional Authority in Cambodia (1992–1993). UNPROFOR, the United Nations Protection Force in former Yugoslavia (1992–1995). ECMM refers to the European Community Monitoring Mission in East/Middle Europe (1991–2007). KFOR is the Kosovo Force (1999–2000), and ISAF the International Security Assistance Force in Afghanistan (2002–2011). See: https://www.nimh.nl/themas/tijdlijn-militaire-geschiedenis-van-nederland/encyclopedie-internationale-vredesoperaties, accessed 27-11-2024.

Initially, 14 veterans were approached, all participating in the first interview. One veteran was excluded for not meeting the moral injury criterion, and another did not participate in the second interview. All participating veterans were male, reflecting that 93.3% of the post-active Dutch veterans are male. The fact that most participants did not identify with a specific religion reflects the Dutch population.^5^ Since the Dutch Ministry of Defence does not administrate ethnicity, we were unable to determine what would constitute a representative sample in this regard and, therefore, did not include this factor in our sampling strategy.

### Data collection

Each veteran participated in two in-depth, semi-structured qualitative interviews (Weiss, 1995). The first interview was conducted at the beginning of the veteran’s engagement with an MC or, for existing contacts, to mark a baseline point in the research process. The initial interview guide consisted of three broad questions, in which we included the time element for our longitudinal approach by asking the veteran to reflect on his current situation and to look backward and forward (Calman et al., 2013). The interview questions were:

- How do you experience moral injury?
- Why did you get in contact with the MC?
- What are your expectations of meeting with the MC?

The second interview was scheduled either at the conclusion of the veteran’s interactions with the MC or as the research approached completion, typically spanning 3 to 16 months, with the majority occurring between 10 and 12 months. The interview guide for this second session included time-based questions and consisted of the following topics:

- How did the conversations with the MC look like?
- How did the MC address the moral dimension of your struggles?
- How did the conversations with the MC aid you in your struggles?
- What changed in your experience of moral injury compared to the first interview?

Veterans provided written consent during the first interview based on an information letter detailing the purpose and design of the research. By doing so, they consented to the data collection through two interviews. Additionally, the veterans figuring in the illustrative cases described below were involved in the writing process and gave consent for the (translated) narratives we present. All participants had the right to withdraw their consent at any point during the research project without needing to provide a reason.

Interviews were conducted at locations preferred by the veterans—mostly their homes, but sometimes at a veteran meeting house or a military barracks—and lasted between 60 and 180 min. All interviews were conducted by the first author, who took special care to establish rapport with the veterans, considering differences in gender, professional background, and sometimes also age (Duncombe and Jessop, 2024). The interviews were all conducted in Dutch.

The veterans frequently used sayings, metaphors, and expressions to describe their experiences, meaning translating the narratives and quotes posed a challenge. We sought assistance from a bilingual Dutch-English speaker to ensure accurate translations of certain expressions.

### Analysis

Longitudinal studies often require multiple analysis methods (Thomson and Holland, 2003). In our study, we used (1) a theoretically informed longitudinal analysis (McLeod, 2003) to be able to look at the development of the experience of moral injury over time and (2) an inductive, thematic cross-sectional analysis to gain deeper insight into the processual experience of moral injury at the time of the first interview and second interview (Thomson and Holland, 2003).

#### Analyzing the experience of moral injury at the initial interview

To start, the 1st and 2nd authors read the interview transcripts multiple times to familiarize themselves with the data (Braun and Clarke, 2012). After that, we conducted an inductive thematic analysis of each interview transcript, directed at gaining a deeper insight into the experience of moral injury at the first and second interviews. We utilized open coding to identify recurring patterns across the dataset (Braun and Clarke, 2012), employing the qualitative data analysis software Atlas.ti to facilitate the coding and organization of themes. Through the inductive thematic analysis of how veterans experience moral injury, we identified 202 quotations and 19 initial codes. These initial codes provided a broad picture of the lived experience of moral injury, including personal struggles such as negative thoughts and emotions, suicidal thoughts, a loss of trust, or the need to be in control. Additionally, codes such as ‘problems of dealing with contemporary happenings,’ ‘problems of dealing with injustice,’ and ‘feelings of guilt toward specific persons’ indicated the relational and social dimensions of the struggles. We clustered the 19 initial codes into three overarching themes, which represent three distinct moral questions underlying veterans’ experiences of moral injury.

#### Analyzing the experience of moral injury at the second interview

The inductive thematic analysis was used to gain insight into what changed in the veterans’ experience of moral injury at the time of the second interview, which resulted in 139 quotations and 25 initial codes. The high number of initial codes says something about how person-specific the value of meeting with an MC may be, but it also resulted from the choice to recognize different ways of expressing certain values in the initial stages of the inductive coding process. For example, we coded ‘feeling heard’ and ‘feeling understood’ as different initial codes and did the same for ‘a place to ventilate thoughts’ and ‘a place to exchange thoughts.’ By focusing on what veterans said about how the conversations with the MC helped them in dealing with their moral struggles specifically, we clustered the initial codes into five types of change that occurred in the lives of the veterans at the time of the second interview.

#### Longitudinal analysis - synthesizing framework

After conducting an inductive thematic analysis, we developed a synthesizing framework (*see* *Table* *2*) for the third step, the longitudinal analysis. This framework allowed us to synthesize the data collected from both interviews for each individual veteran. The questions listed above each column directed our data analysis, leading to brief interpretative notes written by the first author and discussed with the second author. Every row culminated in a ‘case profile’ for a specific veteran, illustrating the changes and continuities in their experience over time (Thomson and Holland, 2003).

**Table 2:** A framework longitudinal analysis.

Veteran	How does the veteran experience moral injury?	Why did the veteran contact the MC, and what were his expectations?	How did the MC address the moral dimensions of their struggles in the conversations?	What changed in the experience of moral injury compared to the first interview?	Case profile
Name	Interpretative note	Interpretative note	Interpretative note	Interpretative note	Narrative of change based on the interpretative notes

#### Abductive analysis of group-wide findings on characteristics of moral learning

In the last step of our analysis, we compared the individual case profiles to search for recurring patterns within the participant group regarding how the veterans experienced support from MCs when dealing with moral injury (Calman et al., 2013). The analytical findings were then interpreted through the lens of moral learning, based on the writings of Dewey (1922, 1932, 1938a, 1938b). This is referred to as an abductive approach (Vila-Henninger et al., 2024) and means that, in this case, the results of the analysis were confronted with the writings of Dewey to reach an even higher level of interpretation.

## Results

We present our findings along three lines. First, we describe how the veterans in this study perceive the moral dimensions of their struggles with moral injury. Second, we focus on the process level of the engagement between veterans and MCs. Here, we present the characteristics of the patterns of change concerning how veterans deal with their moral struggles over the period of this study. We characterize this as a process of moral learning and illustrate these findings with three illustrative cases from the data. Third, we elaborate on the veterans’ views of what has changed in their lives and how they deal with their moral struggles due to engaging in one-on-one conversations with the MCs.

### Moral injury experienced as lingering moral questions

In the interviews, veterans frequently articulated explicit moral concerns. We found three types of moral questions underpinning the veterans’ daily experiences of moral injury.

#### Moral questions about mission-related actions

The first type of moral questions revolves around mission-related action questions, such as *“Did I do good?”* and *“Did I do enough?”* These questions relate both to specific actions taken—or not taken—during missions and extend to broader concerns about whether the mission as a whole had any meaningful impact on the situation at hand:

Freek testifies (interview 1): *“He would come in the morning to help us load some stuff. I promised that he would receive some money for doing that. But because of the bombing, that never happened… I do not know what happened with the boy. Often, I wonder, ‘Why?’ [long silence] Why? [long silence] Do I feel guilty for not having been able to meet him? Perhaps he overslept and never arrived for the meeting.”*

Koen explains (interview 1): *“I became very anti. Especially when I was watching the news… and I thought, ‘Hey, I was walking there, and now I see that the Taliban rules again and that they are killing people…’… that made me wild with anger… and then I thought, ‘what sense did it make that I was walking around there all that time?”*

#### Moral questions about the ‘good life’

The second type of question relates to the concept of a good life. These questions include personal reflections, such as *“Can I still live a good life?”* and broader concerns about humanity, such as *“How can I see human goodness again?”*

Frank testifies (interview 2): “*I see a lot of things happening in our society, and then I wonder, ‘What are we doing?!’ Things will end wrong. I have to do something about that.”*

Veterans describe a heightened sensitivity to situations of injustice, ranging from minor incidents, such as someone cutting in line at the supermarket, to broader geopolitical crises, like the recent attacks on Ukraine. This heightened sensitivity often coincides with an overwhelming sense of responsibility. Veterans not only directly reported this in their interviews, but it was also something that became evident in the first author’s reflective notes, where she described receiving numerous messages from veterans while making the arrangements for the interview. These messages often focused on ensuring her well-being, including thoughtful gestures like asking her how she prefers her tea (black or fresh ginger, with or without lemon?) even before arriving for the interview.

#### Doubts about the ethical conduct of the military apparatus

The third type of moral question relates to doubts about the ethical conduct of the military apparatus. These doubts include concerns about the construction and implementation of UN missions or about veterans’ relationships with the Dutch Ministry of Defence. Veterans ask why they continue to feel loyal to an organization that, in their view, has shown them so little loyalty in return.

Bart explains (interview 2): “*I feel guilty because there is some kind of moral vacuum within the organization. When policy only facilitates the minimum amount of training and resources to avoid doing anything unnecessary. Then, I wonder, ‘Why did things turn out the way they did?’ ‘Could I have changed anything?’ Ultimately, you have to carry this burden alone because these questions will never be answered.”*

We observe that these three types of questions—about mission-related actions, the good life, and the ethical conduct of the Dutch Ministry of Defence—often intersect and reinforce one another. For example, doubts about the ethical conduct of the military can negatively influence veterans’ trust in and outlook on society. Conversely, a deteriorating trust in society can exacerbate their sense of betrayal by the Dutch Ministry of Defence.

### The cases of Jan, Joris, and Simon to illustrate dealing with moral injury as a process of moral learning

Having identified three types of moral questions underpinning veterans’ lived experiences of moral injury, we now turn to the question of how living with these lingering moral questions evolves while meeting with the MC. We illustrate our findings with the help of three exemplary cases representing the three types of moral questions. The veterans in these cases are struggling primarily with one type of moral question. This does not necessarily represent the whole group of veterans in our study but allows us to exemplify how each of the different moral questions evolves (Flyvbjerg, 2006).

Let us first present the three veterans featured in the illustrative cases. Jan participated in UNPROFOR, serving on three missions in 1992 and 1993, and at the end of 1994, as a member of the Dutch Battalion III in Srebrenica. He worked as a medic, responsible for setting up and coordinating on-site medical care. He engaged in conversations with a Catholic MC, primarily to share his doubts about his participation in Dutch Battalion III. Joris also participated in the peacekeeping mission in former Yugoslavia, UNPROFOR, and sought support from a humanist MC after undergoing 7 years of trauma therapy. The moral questions he struggles with primarily concern goodness in himself and goodness of humanity in general. Simon joined the army in 1997 and embarked on his first mission to Kosovo in 1999. He subsequently served in Afghanistan in 2007 and 2008/2009 as a technician with the medical team. During these years, Simon worked under various short-term contracts. He engaged in conversations with the same MC as Jan did. They mainly discussed his questions about the ethical conduct of the Dutch Ministry of Defence.

#### Moral learning is prompted when coping mechanisms fail

The first pattern we identify is that the moral learning process veterans engage in, starts out of necessity. Eleven out of twelve veterans expressed that they did not seek help until their coping mechanisms were exhausted. In five cases, this was due to extremely negative thoughts and the impossibility of connecting with the people around them. Six veterans explained that they only sought help when they only sought help when there were physically and mentally completely exhausted. The cases illustrate this:

##### Jan being triggered to address feelings of doubts and shame around the topic of recognition

Jan says while working as a medic during the UNPROFOR mission, he felt “*like a duck to water*”: he thrived in high-stakes situations and enjoyed organizing comprehensive medical care systems. However, after returning from Srebrenica, Jan faced numerous challenges. The adrenaline of deployment was gone, leaving him with time to reflect on his experiences over the past 3 years. He felt a strong desire to return to “his war” and found reintegrating into civilian life difficult. Simultaneously, news broke of the genocide in Srebrenica, which was widely reported in the media. The role of Dutch Battalion III was portrayed very critically, as the question was raised as to why they could not prevent the genocide. Jan felt abandoned by the Dutch Ministry of Defence, which did not defend Dutch Battalion III then. To avoid the relentless media coverage and his internal doubts and struggles, Jan threw himself into work. Until 2018–2019, his life was focused on physically exhausting himself. This pattern continued until he became so exhausted that he could no longer read. After several medical checks, it was determined that his symptoms were related to his participation in UNPROFOR, and he was referred to specialized trauma therapy. Jan explains that Eye Movement Desensitization and Reprocessing (EMDR) treatments helped him manage his memories. However, when the topic of recognition surfaced, particularly when the Dutch prime minister apologized in 2022 and awarded medals, Jan realized that he did not feel this was just: “*How can you feel good and pleased about something that ended like this?”* He realized that he had been carrying these thoughts and feelings of shame as a heavy burden all this time. It was impossible for him to separate his own memories and experiences from the media portrayals of the mission. Not a day passed without these thoughts crossing his mind. To address these issues, his case manager suggested contacting an MC who is working in his region. This MC has a Catholic affiliation.

##### Joris’ need to address deep feelings of guilt and shame

Joris explains that although trauma therapy helped reduce his re-experiences and taught him how to identify and manage his triggers, his problems still worsened over time. He lost his sense of control, struggled with paranoia, and experienced deep feelings of guilt and shame. Therapies like EMDR did not help alleviate these feelings, and because his struggles persisted, Joris’s case manager suggested reaching out to a humanist MC.

##### Simon’s search for recognition on whether the Dutch Ministry of Defence had treated him fairly

Simon’s reason for seeking contact with an MC also came from physical exhaustion. He explains that his situation culminated in a physical collapse: *“Last year I woke up in the emergency room, the year before in an ambulance… The pain was so intense that I could only vomit until I received a morphine injection to relieve the tension in my back.”* His general practitioner referred him to mental health services, linking the intense back pain to stress. Simon felt uncomfortable discussing his military past with standard mental health services, so he sought help from the Netherlands Veteran Institute and was referred to PTSD treatment. The problem was, however, that he had completely blocked out everything that had happened during the different missions in which he participated: *“My problems pile up, and I, figuratively speaking, throw them over a big wall. Until now, I have always managed to leave my problems there.”* Mission-related experiences only emerged through intense nightmares that severely disrupted his sleep. Although he did not recall specific details, he knew that his nightmares were mission-related because others had heard him talk about them during these nightmares. His social worker recognized that Simon was not only struggling with PTSD but also with moral questions, particularly about whether the Dutch Ministry of Defence had treated him fairly. She suggested contacting an MC, which Simon agreed to, hoping that the MC could help him clarify his situation: “*If she can help me get my story straight and ensure the right people hear it, that would be great. I hope it will help resolve my situation.”*

The lens of moral learning, based on Dewey’s writings, helps to illuminate that even though in all cases someone else, a case manager or social worker, suggests reaching out to an MC, the veterans’ agreement to act on this suggestion and engage in reflection with an MC was a matter of necessity.

#### Moral learning through reflecting as equal partners on moral questions

The second pattern we identify is that the kind of learning these veterans engage in does not result from direct instruction. MCs do not tell the veterans what a good life entails and do not aim to reverse negative thoughts, such as feelings of guilt, in a directive way. The longitudinal analysis shows that veterans and MCs exchange thoughts on mission-related events and/or ongoing daily struggles. Moreover, these conversations happen without an agenda set beforehand and in a reciprocal way. Veterans express that they almost felt like talking to a friend since MCs sometimes shared some of their own struggles and did not approach them as clients.

The lens of moral learning enables us to comprehend these relational and reflective interactions as experiential forms of moral learning. Furthermore, it demonstrates that these discussions are nodes in a more extensive process that unfolds over time within the lives of the veterans. As previously stated in the introduction, moral learning does not happen overnight. Only when these novel modes of action and interpersonal engagement are met with responsive reactions from others, they attain significance and can evolve into new habits. The MC facilitates this learning process by enabling reflection and acting as one such responsive other. Notably, the MC is engaged in this process as an individual *and* a representative of the Dutch Ministry of Defence. However, the process of acquiring new habits is not solely contingent on the MC; it depends on the potential for these new behaviors to become integrated into each veteran’s social and situational environment. This process takes a considerable amount of time; in only four cases, the conversations between the MC and the veteran concluded within a year. The cases illustrate this further:

##### Addressing Jan’s struggles around the topic of recognition

The interviews with Jan reveals that the Draaginsigne Gewonden (DIG) ceremony—a Badge for the Wounded, equivalent to the American Purple Heart—was a focal point in their meetings. However, the exact content of their discussions was not predetermined. Jan explains: “*We take a seat, have a cup of coffee, and then it happens*.” The main topic they addressed was his struggle to feel good about his participation in Dutch Battalion III. Even though he provided much humanitarian aid, such as medical care for childbirths with complications, he still could not reconcile feelings of pride or reward for “*a mission that failed totally and had as its result – next to all the people that got wounded through the violence of the war – 9.000 concrete people that got murdered.”* Jan explains that his conversations with the MC served as a mirror, helping him break through a negative thinking spiral. They enabled him to view his actions from an outsider’s perspective and separate his own efforts from the mission at large: “*My actions and my performances during the mission have always been optimal. Even if I had done things differently, the course of history would not have been different*…” This realization alleviated much of the shame he felt and was a crucial first step in addressing his moral struggles. The presentation of the DIG itself served as a crystallizing moment in this process when the badge for the wounded was presented to Jan by his former commandant in the presence of the MC and his family and friends. Jan shares that this was the first time he heard his superior say he had done all he could. Compelled by the situation, Jan had written down his thoughts in preparation for his speech. This was the first time he sat down, wrote down the whole narrative, and shared these feelings with his family and friends. Jan explains this event’s meaning: “*The DIG is only a piece of metal, but that piece of metal is symbolic. I think through the DIG, my acquaintances were acknowledged, and perhaps they also understood or accepted me a little better. I feel that everything was harmonized in the handing over of the medal.”*

##### Reflecting with Joris on his low self-esteem and his moral struggles in daily life

Joris and the MC met once every 6 weeks at a veteran meeting house that Joris frequented. Just as with Jan, there is no set agenda; Joris decides what is discussed: *“We take a seat, and we start to talk. We might discuss nothing special in one meeting, while we have deep conversations another time. [It depends on] how I am feeling at that moment*.” Their conversations often revolved around his daily struggles to live a good life. Joris particularly struggles with the question, “Am I a good person?” He has very low self-esteem and finds looking at himself in the mirror difficult. Moreover, situations of injustice—minor or major—are significant triggers for him. These struggles hinder his ability to connect with others, as he fears hurting them. In the conversations, Joris and the MC touched upon such existential matters, but they also addressed specific instances from his daily life, such as Joris’s reactions to family quarrels or a visit to a busy IKEA. Notably, the morally injurious events themselves were never addressed during the 2 years that Joris and the MC have been meeting. Joris states, “*I’ve been addressing these incidents in therapy for the last seven years. I’ve closed the door on that chapter in my life.”*

Nevertheless, Joris says he has benefited greatly from his talks with the MC. Joris explains that with the MC, he can be open, vulnerable, and honest about his daily struggles. He appreciates that the MC does not watch the clock or tries to label his experiences; he simply listens. Joris: “*You just meet as two human beings*.” This approach makes Joris feel accepted and creates a safe space for him to share more of his struggles. It is not only the relational dimension that Joris values in his conversations with the MC. He also appreciates that the MC is part of the military and has been on a mission. Because of this shared background, Joris did not feel the need to explain certain behaviors, which allowed more space to discuss the issues that truly matter to Joris.

##### Exploring Simon’s ambiguous relationship with the Dutch Ministry of Defence

Simon and the MC began meeting monthly, in parallel with his PTSD therapy and the process of making the case with his social worker regarding his delayed dismissal. Simon found it extremely helpful to discuss with the MC whether his expectations of the Dutch Ministry of Defence were justified: “*The MC is part of the military, so she knows how this world works. Therefore, I can exchange thoughts with her, like, ‘Is it too much to ask for a job?’*“Beyond addressing practical concerns, the MC also grasped his ambiguous feelings of loyalty toward the military. Simon explains that, for him, it is not all about the money. It has much more to do with his relationship with the Dutch Ministry of Defence. Simon is profoundly hurt and angry with the military but remains loyal. The core of his struggle is to understand why he continues to feel loyal to an organization that seems to show him so little loyalty in return. Simon initially had little trust that someone from the Dutch Ministry of Defence would genuinely listen to him instead of sweeping things under the rug. However, the MC did listen, making him feel heard and seen. This opened the door for him to recognize himself as a victim of certain situations.

#### Moral learning as a transformative process, including the moral understanding of actions, the self, and others

Dewey’s writings illuminate that moral learning processes are transformative. It can fundamentally change someone’s moral outlook on specific actions, the self, and others. As written in the introduction, the concept of habits serves as a tracer to observe if moral learning leads to a transformation of someone’s selfhood and worldview. Therefore, in the analysis, we not only marked quotes in which veterans expressed that they gained new perspectives, but we also examined new habits that are indicative of such transformative changes. Seven out of twelve veterans indeed expressed that their changed perspectives on themselves, the mission, and the world opened up new ways of relating to others, seeing their selves, and acting in the world.

We found five types of change in veterans’ experience of moral injury. First, conversations with MCs allow veterans to share their stories, thoughts, and worries about mission-related events or issues in their daily lives and current society. Given that veterans often reported feeling isolated in their struggles and having no opportunity to share their stories before meeting with the MC, this represents a change in itself and not merely a condition for change. Furthermore, we posit that the ability to narrate their experiences, reflections, and concerns represents a more profound shift than merely disclosing a previously latent story. Veterans frequently indicated that they had to learn to narrate these accounts. They also observed that by learning to recount their experiences, they were able to gain control over the fragmentary thoughts that had previously exerted a negative influence on their self-image and worldview.

Second, conversations with an MC help veterans to grow personally. They gain greater self-confidence, feel seen and heard, and experience a stronger recognition for their struggles and contributions during their missions. Joris, for example, stated that he now feels self-secure enough to start tinkering with cars at the veteran meeting house, which, in return, also strengthens his growing self-confidence.

Third, veterans are able to better understand and accept certain events, which may lead to forgiveness—of others or of themselves. As the case of Jan illustrates, he was able to reduce his major involvement in several Non-governmental organization (NGOs) as he now feels he has fulfilled his moral obligation.

Fourth, the conversations also help veterans feel a stronger connection with loved ones, including partners and children, and with the world around them. Finally, conversations with MCs allow veterans to critically engage with the Dutch Ministry of Defence. As Simon’s case illustrates, this can sometimes mean taking steps in a call for justice. This, in turn, may alleviate some of the tension stemming from simultaneous feelings of loyalty and betrayal.

##### Jan’s moral perceptual change in understanding the mission, the self, and his need to do penance

Jan’s conversations with the MC forced him to reflect more deeply on the struggles he had been suppressing for the last 28 years. In Jan’s case, the DIG ceremony served as a crystallizing moment in this relational and situated form of learning, in which three perspectives—his own, his commandant’s, and that of the MC—helped him realize that the heavy burden he was carrying is, in fact, a collective one. This profoundly changed his moral outlook on his participation in the mission and himself. He now feels less obliged to continue with full force in all the different aid organizations he is involved in. Previously, he felt a moral obligation to help, such as supplying medical materials for Ukraine. He will continue to do this, but he no longer feels the moral pressure to the same extent and can limit his involvement: *“It has been enough. I do not feel the obligation to… do good… to suppress my feelings of shame.”*

##### Joris’s newly acquired ability to break negative thought spirals and trust others

As Joris discussed his daily struggles, especially regarding his low self-esteem, the MC pointed out the changes he noticed compared to 2 years ago. The MC’s feedback on his progress helped Joris break out of his negative spiral and recognize the progress he is making. Joris explains that while the mirroring remarks of the MC are perhaps small interventions, they helped him enormously:

*I always have to process a meeting [with the MC], and when I talk about it later with my wife, she gets a bit upset that I accept his [the MCs’] feedback while not listening to hers. She mentions similar things, but she phrases them differently. She always says that my brain is good and I should trust myself more. He says essentially the same thing, but he does not tell me directly—he shows me. For instance, I might show him a WhatsApp conversation that bothers me, and he says, ‘It’s just a WhatsApp message. Let it go. I see what you did, and it’s right. Just do what you feel is right, and that’s fine.’ This helps lift me out of my negative thought spiral. That’s what he does.”*

Joris recognizes that the MC and his wife convey similar messages, but their approaches differ. While his wife tells him that he should trust himself, the MC uses concrete examples of Joris’s actions to reflect upon. This helped him better acknowledge the progress he has made over the last 2 years but also gave him confidence that it is normal in human relationships to sometimes hurt each other or make mistakes. Joris has discovered a newfound trust and self-confidence through the meetings with the MC, allowing him to connect more deeply with his emotions and improve his relationships, notably his marriage.

##### Simon’s new-gained ability to be critical on the Dutch Ministry of Defence

During the second interview, Simon explained that he now understands that being a victim of certain events does not define his entire identity or indicate weakness. He has gained a new understanding of what being a victim means, which has enabled him to see himself as a victim of certain happenings. This was crucial for him in seeking and accepting help. He shares that the wall he built to hide his problems remains largely intact and that it will only come down when he feels sufficiently safe. Although he has not yet reached that moment of complete safety, he is progressing. When, in the interview, the first author asked Simon to explain how he understands progress, Simon referred to specific changes in his behavior. He is gaining new habits, which are as concrete as making a document to fight delayed dismissal. He can only do this because he now feels the space to be critical of the Dutch Ministry of Defence. He is also sleeping better, with nightmares occurring only every 2 weeks instead of almost nightly. These new habits allow him to start living a life outside the confines of his home. By the time of the second interview, Simon planned to return to Kosovo to celebrate the 25th anniversary of its liberation. Reaching out to him while writing this article revealed that this return trip was significant for his recognition from local civilians and the Netherlands Veteran Institute. He acquired a veteran uniform and participated in various festivities, which helped him view himself as a veteran and appreciate his work, despite his lingering resentment about how the Dutch Ministry of Defence treated him afterward.

## Discussion

Our study offers further insights into lived experiences of moral injury, focusing on its moral dimensions and the challenges of living with these struggles in daily life—often decades after the morally injurious events occurred. Our findings show that moral injury not only reflects on mission-related events but also profoundly impacts veterans’ perceptions of goodness in themselves, others, and the world. These prolonged effects were noted as early as Shay (1994)'s observation of moral injury, leading to a “*persistent shrinking of soldiers’ temporal, social, and moral horizons*” (p. 176). Our research is in alignment with his findings that the context of a war situation has a significant impact on the perception of betrayal, in our case, by the Dutch Ministry of Defence and the Dutch government in general.

Furthermore, the experience of betrayal has been shown to have a significant negative impact on an individual’s overall trust in society (Shay, 2014). Similarly, Bica (1999) highlighted how moral injury erodes meaning and connection in veterans’ current lives. Recent empirical work by Molendijk et al. (2018) further elaborates on these aspects. She highlights that moral injury results from morally ambiguous situations that, precisely because they involve moral dilemmas that cannot be resolved, simplified, or compartmentalized, keep haunting somebody, also influencing one’s general outlook on the goodness of both oneself and the world (Molendijk, 2020, p. 171).

Additionally, contemporary research links moral injury to identity, highlighting how moral injury is reflected in one’s character and identity (Atuel et al., 2021; Grimell and Atuel, 2023; Richardson et al., 2022). This indicates how moral injury can cause veterans to feel “broken” as human beings, perpetuating a “*lifelong existential rollercoaster*” (Grimell, 2023b, p. 9). The three types of moral questions that we found to underpin veterans’ experiences of moral injury contribute further empirical evidence to the existing literature on the long-term effects of moral injury.

At the core of our study is a detailed exploration of how veterans experience how MCs address their moral struggles, reflecting the complex ways in which moral injury shapes veterans’ daily lives. First, veterans highly value the adaptability of MCs, who do not follow a fixed agenda but instead are attuned to the specific struggles the veteran is facing. The MC serves as a companion for the veterans, offering a listening ear and acting as a fellow traveler in their pursuit of a fulfilling life. By facilitating reflection on the ongoing process, the MC may influence the veterans’ perspectives and outlook on life. This perspective challenges the structured, goal-oriented approach of existing interventions like *Building Spiritual Strength* (Usset et al., 2021), *Acceptance and Forgiveness Therapy* (Pernicano et al., 2022), and *Pastoral Narrative Disclosure* (Carey and Hodgson, 2018). While these interventions incorporate flexibility (e.g., revisiting steps as needed), they are nonetheless built around predefined care goals. In contrast, our study highlights that in MC practice, chaplains act as conversation partners, offering existential expertise and military, cultural competence attuned to individual veterans’ unique and dynamic needs. While this outlook on MC practice has been noted by MCs themselves (e.g., Grimell, 2023a; Pleizier and Schuhmann, 2022), this study shows that veterans value this aspect of MC practice.

Second, while existing chaplaincy interventions often center on reflecting upon morally injurious events (Fleming, 2020; Usset et al., 2021; Pernicano et al., 2022) or at least use these events as a starting point (Carey and Hodgson, 2018), our study raises questions about the centrality of the morally injurious events in addressing moral injury. Our findings confirm the importance of reviewing morally injurious events to find meaning, accept these occurrences, and potentially forgive others or oneself. However, we did not find that these events necessarily need to have a central place in chaplaincy care. For instance, one veteran, Joris, explicitly avoided reflecting on these events but still found great value in meeting with the MC to deal with his moral struggles. Our data suggest that reflecting on questions concerning morally injurious events is only one way MCs support veterans struggling with moral injury. For veterans, the MC’s support in dealing with questions about the good life and the conduct of the military may be just as important or even more important.

Third, our study highlights the importance of engaging with seemingly mundane, everyday issues when addressing the moral dimensions of veterans’ struggles. This approach contrasts with the emphasis on grand concepts such as “forgiveness,” “acceptance,” “reconciliation,” “restitution,” and “vindication,” which are commonly highlighted by authors when discussing care in the context of moral injury (Koenig et al., 2023). While our study shows that veterans sometimes recognize the value of discussions with MCs in achieving these outcomes, such topics are not explicitly covered in their conversations with MCs. Instead, veterans in our study primarily value the opportunity to reflect on their ongoing, daily moral struggles. These larger concepts become meaningful and relevant in their lives through reflection on everyday issues. The three cases illustrate that just as veterans’ perceptions of themselves and the world change after experiencing inhumane events, achieving moral re-orientation also requires active, lived, and relational engagement with the world.

Moreover, it is important to note that the moral learning processes of the veterans sometimes involve a decrease in efforts to seek forgiveness. MCs can help veterans feel that they have atoned (fulfilled their penance) and restored themselves. This perspective offers a fresh view on the ‘renewal’ phase in the *Pastoral Narrative Disclosure* intervention, which aims to encourage veterans to engage in meaningful activities (Carey and Hodgson, 2018). Our study shows that sometimes, it may be necessary to help veterans feel that they have fulfilled their penance.

These three findings about the value veterans place on one-on-one conversations with MCs—the importance of attuning to the individual rather than pursuing fixed care goals, the need to address moral questions beyond the injurious events, and the focus on daily life occurrences— provide crucial insights into how MCs can support veterans in their moral struggles. Our findings align with the work of other authors who have underlined that living with moral injury involves finding meaning in both the morally injurious events and our world (Kopacz et al., 2019; Usset et al., 2024). It includes finding moral reorientation (Molendijk, 2020), learning to navigate between different moral perspectives related to their various roles in life—as veterans, civilians, or perhaps parents (Grimell and Atuel, 2023), and gaining new meaning through identity transformation and reconstruction (Grimell, 2022). Our proposal to view the engagement between veterans and MCs as a process of experiential moral learning encapsulates these views on dealing with moral injury, given our earlier explanation of moral learning as a process of moral reorientation, meaning-making, and identity transformation. Moreover, framing moral injury recovery as a learning process avoids rigid treatment protocols and emphasizes the importance of attuning to the individual’s moral framework (Usset et al., 2024). This perspective also resonates with the view of moral injury as providing insight into and challenging societal values and practices (Wiinikka-Lydon, 2018, 2022; Antal, 2017; Antal et al., 2023). By emphasizing the relational and social nature of moral learning processes in relation to moral injury, moral injury can be understood as a call for broader societal learning. In this view, learning from moral injury appears as a shared societal endeavor, shifting responsibility from veterans to the broader society (Kramer and Molendijk, 2023).

### Limitations

The geographical setting of this research introduces certain limitations. The Netherlands is one of the most secularized countries in the world, with less than half of its population identifying with a spiritual community and only 12% attending religious services regularly.^6^ Reflecting this secularism, over half of the veterans in our study reported having no affiliation with religious communities. This context raises questions about the transferability of these findings to other national and cultural contexts: are these findings particularly relevant to how MCs address the moral injury of veterans in other secular settings? How do they apply to MCs’ work in more religious contexts? Addressing these questions could be a valuable direction for future research.

An additional limitation of our study is its sample, which consists solely of veterans who have reported benefiting from one-on-one conversations with MCs. This focus aligns with our study’s objective: to provide insight into what veterans value in these conversations rather than exploring why some veterans may choose not to continue discussions with an MC or engage with one in the first place. Another limitation is that the empirical scope of this article does not allow for an extensive theoretical elaboration on the concept of moral learning and its integration into theoretical discourses about the moral dimensions of moral injury. Future theoretical research could significantly enhance our understanding and conceptualization of addressing the moral dimensions of moral injury by supporting moral learning.

## Data Availability

The data that support the findings of this study are available from the corresponding author, LM, upon reasonable request.

## Footnotes

^1^De Veteranennota 2023-2024| https://www.rijksoverheid.nl/documenten/rapporten/2024/06/14/veteranennota-2023-2024 accessed 29-11-2024.

^2^Religieuze betrokkenheid in Nederland|CBS https://www.cbs.nl/nl-nl/longread/statistische-trends/2023/religieuze-betrokkenheid-in-nederland?onepage=true accessed 29-11-2024.

^3^FS6-Posttraumatische-stressstoornis-na-uitzending1.pdf accessed 04-12-2024.

^4^These veterans engaged in conversations with 6 different MCs, which are all the MCs who are involved in one-on-one spiritual counselling for post-active veterans in the Netherlands. The MCs represent the following denominations: humanist (n = 2), Catholic (n = 2), and Protestant (n = 2), with professional experience ranging from 7 to over 30 years.

^5^https://www.cbs.nl/nl-nl/longread/statistische-trends/2023/religieuze-betrokkenheid-in-nederland?onepage=true. Accessed 30-09-2024.

^6^https://www.cbs.nl/nl-nl/longread/statistische-trends/2023/religieuze-betrokkenheid-in-nederland?onepage=true accessed 26-08-2024.
